# Targeting Syk in Autoimmune Rheumatic Diseases

**DOI:** 10.3389/fimmu.2016.00078

**Published:** 2016-03-07

**Authors:** Guo-Min Deng, Vasileios C. Kyttaris, George C. Tsokos

**Affiliations:** ^1^Key Laboratory of Antibody Techniques of Ministry of Health, Nanjing Medical University, Nanjing, China; ^2^State Key Laboratory of Reproductive Medicine, Nanjing Medical University, Nanjing, China; ^3^Beth Israel Deaconess Medical Center, Harvard Medical School, Boston, MA, USA

**Keywords:** Syk, autoimmune disease, Syk inhibitor, IgG, Fcgamma receptors

## Abstract

Spleen tyrosine kinase (Syk) is a member of the Src family of non-receptor tyrosine kinases, which associates directly with surface receptors, including B-cell receptor and Fcγ receptor, and is involved in a variety of signal transduction pathways. Rheumatoid arthritis (RA) and systemic lupus erythematosus are autoimmune diseases in which autoantibodies, immune complexes, and autoreactive T cells account for the expression of tissue inflammation and damage. Syk inhibitors efficiently suppress RA in patients albeit in the expression of unwanted side effects, including gastrointestinal effects, hypertension, and neutropenia. Syk inhibitors also inhibit clinical manifestations in lupus-prone mice. Here, we review the evidence that supports the use of Syk inhibitors to treat rheumatic and other autoimmune diseases.

## Introduction

Spleen tyrosine kinase (Syk) is a cytoplasmic protein-tyrosine kinase and a member of the Src family of non-receptor tyrosine kinases (1). The Syk protein contains a pair of Src homology 2 (SH2) domains at the N-terminus that are joined to each other by linker A and are separated by a longer linker B from the catalytic domain (2, 3). Syk is activated when the tandem SH2 domains are engaged or when tyrosines participating in the linker–kinase sandwich become phosphorylated. SH2 domains are structural motifs that bind phosphotyrosine to enhance protein–protein interactions (4, 5). These high affinity Syk-binding sites are known as immunoreceptor tyrosine-based activation motifs or ITAMs, which are located in many important receptors (6). Syk physically docks to the doubly phosphorylated ITAM *via* its tandem SH2 domains in a head-to-tail orientation. Conformational changes disrupt the “linker–kinase sandwich” and activate the enzyme (7).

Spleen tyrosine kinase catalyzes the phosphorylation of proteins on tyrosines located at sites (8). Signals are further transmitted from the Syk-receptor complex through the phosphorylation of adapter proteins, such as BLNK/SLP-65, SLP-76, and LAT (3, 9). These phosphorylated proteins serve as scaffolds to which effectors dock with SH2 or other related phosphotyrosine-binding motifs. Effectors include members of the Tec-family of tyrosine kinases, lipid kinases, phospholipases, and guanine nucleotide exchange factors that further propagate the signal allowing for the activation of multiple pathways, including PI3K/Akt, Ras/ERK, PLCγ/NFAT, Vav-1/Rac, and IKK/NFκB (2, 3).

**Figure 1:**
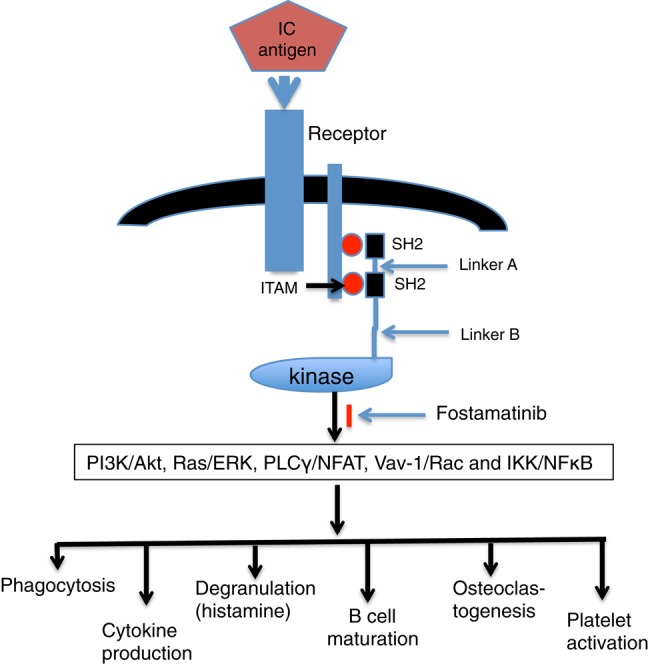
**Engagement of Syk and downstream effects**. Following aggregation of FcR by immune complex (IC), the phosphorylation of ITAM tyrosine leads to the recruitment of Syk to the receptor in an interaction mediated by its tandem pair of SH2 domains. Active Syk initiates signaling pathways of PI3K/Akt, Ras/ERK, PLCγ/NFAT, Vav-1/Rac, and IKK/NFκB and then generates downstream effects, such as phagocytosis, cytokine production, degranulation, B-cell maturation, osteoclastogenesis, and platelet activation.

Spleen tyrosine kinase is widely expressed in the hematopoietic system and is involved in a variety of signal transduction pathways, including receptor signaling in mast cells, monocytes, osteoclasts, and T, B cells (10–16) (Figure 1). In this review, we discuss the role of Syk in Fcγ receptor (FcγR) signaling and the effect of Syk inhibitor in treatment of autoimmune diseases.

## Syk and IgG/Fcγ Receptor Signaling Pathway

IgG is recognized by FcγR, and IgG–antigen (Ags) complexes bind to FcγR on immune cells to mediate inflammatory immune responses. There are three kinds of FcγR: FcγRI, FcγRIIA, and FcγRIIIA. IgG-binding FcγR induces activation of Syk through ITAMs defined by these receptors (17). Receptor engagement enhances the phagocytosis of IgG-opsonized particles and the production of cytokines, nitric oxide, and reactive oxygen species, which promote the killing of microbes and cause tissue inflammatory damage. Syk-deficient macrophages cannot phagocytose IgG-coated particles, and Syk-deficient neutrophils fail to undergo an oxidative burst in response to the engagement of FcγRs (18, 19). In neutrophils, integrins signal through an association with either FcγR or DAP12, another ITAM-containing accessory protein, and Syk is required for adhesion-dependent activation (20).

## IgG/Fcγ Receptor Signaling and Autoimmune Diseases

Type II and Type III hypersensitivity reactions are mediated by IgG that interacts with bound and soluble Ags, respectively, and are responsible for the inflammation that accompanies many autoimmune diseases.

B and T cells have been shown to exert an important role in the pathogenesis of autoimmune diseases (21). The T cell receptor (TCR) is associated with the CD3 complex, which includes a dimer of ζ chains each of which contains three ITAMs (15). TCR engagement triggers the phosphorylation of ζ chain ITAM tyrosines that leads to the binding of Zap-70. B cells are responsible for production of IgG and are activated through the B-cell receptor (BCR). BCR consists of a membrane spanning immunoglobulin in association with two signaling adaptors: CD79a (Ig-α) and CD79b (Ig-β), each of which contains a single ITAM (2, 3). Syk-deficient mice lack mature B cells (22). Disruption of the Syk gene in DT40 B cells blocks essentially all BCR-stimulated signaling pathways (23).

Systemic lupus erythematosus (SLE) is a chronic autoimmune disease characterized by high levels of autoantibodies and multiorgan tissue damage. The TCR–CD3 complex in SLE T cells is rewired in that the levels of CD3ζ is decreased, and its place is taken by FcγR, which recruits Syk and not Zap-70 as its signaling partner (24). Much of the altered gene expression that characterizes SLE T cells (e.g., increased expression of IL-21, CD44, PP2A, and OAS2) can be induced by the overexpression of Syk in normal T cells (25). High level of autoantibodies in serum and IgG deposition in tissues typify SLE. Circulating immune complexes (ICs) and primarily those formed *in situ* are important in the expression of the inflammatory response (20).

Rheumatoid arthritis (RA) is a chronic autoimmune disease characterized by joint inflammation and bone destruction (26). T cells (especially Th1 and Th17 cells) are important in the pathogenesis of RA (27, 28). Recently, follicular helper T (Tfh) cells, whose primary task is to drive the formation of B cell responses, have been recognized as critical regulators of autoimmunity (29, 30). Levels of pSyk in peripheral blood B cells are preferentially higher in patients with RA compared to healthy subjects. Patients with significantly higher pSyk levels are strongly positive for anti-citrullinated protein antibodies (31). Mice deficient in FcγR or FcγRIII fail to develop collagen-induced arthritis (32), and genetic deficiency of Syk protects mice from autoantibody-induced arthritis (33). The depletion of Syk from neutrophils alone is effective in blocking joint inflammation in autoantibody-induced arthritis (34), and direct injection of naked Syk siRNA into joints inhibits the development of arthritis (35).

Systemic sclerosis (SSc) is a chronic autoimmune disease with a high morbidity and mortality. Skin and organ fibrosis are key manifestations of SSc, and pathogenesis remains unclear (36). Syk inhibitor fostamatinib was demonstrated to limit tissue damage and fibrosis in a scleroderma mouse model (37). It indicates that the Syk pathway appears as a potential molecular target for therapeutic intervention in SSc.

Thrombocytopenic purpura (ITP) and heparin-induced thrombocytopenia (HIT) are autoimmune diseases in which autoantibodies against Ags on platelets result in platelet activation and the opsonization and phagocytosis of both platelets and megakaryocytes by macrophages. Syk inhibitors block IC-mediated platelet activation through FcγRIIA in a mouse model of HIT (38). Fostamatinib (a Syk inhibitor) blocks platelet loss induced by an antibody (Ab) against integrin αIIβ in a mouse model of ITP (39). A Phase II clinical trial in patients demonstrated that fostamatinib can restore platelet counts in approximately 50% of patients with ITP (39).

## Efficacy of Syk Inhibitor on Patients with Rheumatoid Arthritis

A highly specific Syk inhibitor, known as R406, has been shown to block Fc receptor signaling (40, 41). R788 (renamed fostamatinib) is a small molecule, water-soluble prodrug of the biologically active R406 and a potent inhibitor of Syk (42). The small molecule, R406 as well as R788, has been shown to inhibit the development of experimental arthritis (43, 44). In a randomized clinical Phase II trial, fostamatinib when added to background treatment with methotrexate at a stable dose was effective in the treatment of patients with RA (45, 46). Side effects included diarrhea, neutropenia, alanine transferase elevation, and increased blood pressure. Most side effects were associated with the higher doses of fostamatinib. Thus, although fostamatinib is a useful DMARD, its clinical use has been precluded by the recorded unexpected side effects.

## Efficacy of Syk Inhibitor on Lupus MRL/lpr Mice

Increased expression of Syk in SLE T cells affect the expression of a number of enzymes, cytokines, and receptors, which are important in disease pathogenesis, suggesting Syk may become therapeutic target in SLE patients (25). In addition, IgG is involved in the skin and kidney injury in SLE patients (21, 47), and intradermal injection of lupus serum IgG induces skin inflammation (47). The expression of Syk is increased in the skin lesion of lupus MRL/lpr mice (48), and the Syk inhibitor R788 completely abrogates skin inflammation induced by lupus serum (Deng, unpublished data). The Syk inhibitor R788 can prevent skin injury and also suppress established skin injury in lupus MRL/lpr mice. Interestingly, discontinuation of treatment results in extended suppression of skin disease for at least 8 weeks (48). Finally, a Syk inhibitor has also been demonstrated to prevent and improve, if administered after the beginning of the disease, of kidney damage in lupus-prone mice (48, 49).

## Expression of Syk as a Parameter of Pathology in RA and SLE

Spleen tyrosine kinase is expressed in rheumatoid synovium, with activated phosphorylated Syk being differentially expressed between RA and OA synovium (41). Syk activation plays an essential role in TNF-α-induced cytokine production in fibroblast-like synoviocytes and RANKL-induced osteoclastogenesis (3, 41). Expression of Syk is abnormally increased in T cells of SLE patients (24) and skin lesion in lupus-prone mice (48). Expression of Syk is associated with disease progression in lupus-prone mice (48), thus expression of phosphorylated Syk may be worked as a parameter of pathology of RA and SLE.

## Side Effects of Syk Inhibitor Fostamatinib

In the trials of RA patients, side effects of Syk inhibitor fostamatinib (R788) were observed. These side effects include diarrhea, nausea, hypertension, dizziness, headaches, neutropenia, upper respiratory tract infections, and increased serum alanine transaminase (ALT) levels (45, 46, 50). Diarrhea and neutropenia are the two most common adverse events in the overall safety population. These side effects were dose dependent and were often reported with the 150 mg bd dose of fostamatinib. Diarrhea occurred in 6 (13%), 5 (11%), 8 (16%), and 21 (45%) of the patients in the placebo and R788 50, 100, and 150 mg groups, respectively (45, 50). The number of neutrophil returned to normal in all patients within 3–7 days after interruption or reduction of the fostamatinib dose (46). Neutropenia caused by Syk may be by Syk-impairing bone marrow neutrophil release, and concurrent MTX use may also play a role (51). Hypertension was a potential side effect of concern. The increase in blood pressure was observed at month 1 in the fostamatinib groups (45, 50). Increases in blood pressure were more pronounced in patients with existing hypertension at screening or baseline. All cases responded to conventional antihypertensive medication or reduction in fostamatinib dose. It has been postulated that an off target effect on vascular endothelial growth factor receptor 2 (VEGFR) may be responsible for hypertension (52).

## Follicular Dendritic Cells in Autoimmune Diseases

Follicular dendritic cells (FDCs) are unique immune cells that contribute to the regulation of humoral immune responses. FDCs are located in the B-cell follicles of secondary lymphoid tissues, where they trap and retain Ags in the form of highly immunogenic ICs consisting of Ag plus specific Ab and/or complement proteins through Fc and C receptor (53, 54). FDC–FcγRIIB exerts an essential role in mediating IC periodicity, Ag-presentation, inducing germinal center (GC) reaction, and generating specific Ab responses. Binding of ICs to FDC–FcγRIIB induces FDC activation that leads to significant upregulation of FDC–ICAM-1, FDC–VCAM-1, and FDC–FcγRIIB itself (54). IC-bearing FDCs and autoreactive GCs frequently exist in autoimmune diseases (55, 56). Interference with FDC-reticula attenuates autoreactive GC formation, reduces pathogenic auto-Ab titers and memory B cells, and ameliorates arthritis (56–58). It has been recently demonstrated that FDC follicular units develop in RA synovium (56, 59). The high levels of FcγRIIB in FDCs protects the immunogenicity of FDC–ICs by minimizing serious inhibition of B-cell activation upon BCR/FcγRIIB crosslinking (54, 60). Actually, the expression of FcγRIIB is significantly reduced on RA memory B cells and plasmablasts, and these alterations on FcγRIIB are associated with high levels of anti-citrullinated vimentin auto-Abs (61). It is not clear whether Syk inhibitor fostamatinib blocks FDC activation and signal transduction.

## Conclusion

Based on the evidence, Syk exerts an important role in the IgG/FcγR signaling pathway and in the aberrant signaling of SLE T cells. There is ample evidence from the study of human samples preclinical experiments that signaling involving Syk contributes to the pathogenesis of autoimmune diseases. Syk inhibitors efficiently suppress RA in patients albeit in the expression of unwanted side effects and raise platelet counts in patients with immune thrombocytopenia. In lupus-prone mice, systemic administration of Syk inhibitors results in the prevention or treatment of skin and kidney injury. It is hoped that more specific inhibitors of Syk devoid of side effects should prove of great clinical value.

## Author Contributions

G-MD organized and wrote manuscript, GT organized and edited the manuscript, and VK organized the manuscript.

## Conflict of Interest Statement

The authors declare that the research was conducted in the absence of any commercial or financial relationships that could be construed as a potential conflict of interest.
